# Novel proteasome inhibitor ixazomib sensitizes neuroblastoma cells to doxorubicin treatment

**DOI:** 10.1038/srep34397

**Published:** 2016-09-30

**Authors:** Haoyu Li, Zhenghu Chen, Ting Hu, Long Wang, Yang Yu, Yanling Zhao, Wenijing Sun, Shan Guan, Jonathan C. Pang, Sarah E. Woodfield, Qing Liu, Jianhua Yang

**Affiliations:** 1 Department of Neurosurgery, Xiangya Hospital, Central South University, Changsha 410008, P. R. China; 2 The Institute of Skull Base Surgery and Neurooncology at Hunan Province, Changsha 410008, P. R. China; 3 Texas Children’s Cancer Center, Department of Pediatrics, Dan L. Duncan Cancer Center, Baylor College of Medicine, Houston, TX 77030, USA; 4 Department of Ophthalmology, Shanghai Tenth People’s Hospital, Tongji University School of Medicine, Shanghai 200072, P. R. China; 5 Division of Pediatric Surgery, Texas Children’s Hospital Department of Surgery, Michael E. DeBakey Department of Surgery, Dan L. Duncan Cancer Center, Baylor College of Medicine, Houston, TX 77030, USA

## Abstract

Neuroblastoma (NB) is the most common extracranial malignant solid tumor seen in children and continues to lead to the death of many pediatric cancer patients. The poor outcome in high risk NB is largely attributed to the development of chemoresistant tumor cells. Doxorubicin (dox) has been widely employed as a potent anti-cancer agent in chemotherapeutic regimens; however, it also leads to chemoresistance in many cancer types including NB. Thus, developing novel small molecules that can overcome dox-induced chemoresistance is a promising strategy in cancer therapy. Here we show that the second generation proteasome inhibitor ixazomib (MLN9708) not only inhibits NB cell proliferation and induces apoptosis *in vitro* but also enhances dox-induced cytotoxicity in NB cells. Ixazomib inhibits dox-induced NF-κB activity and sensitizes NB cells to dox-induced apoptosis. More importantly, ixazomib demonstrated potent anti-tumor efficacy *in vivo* by enhancing dox-induced apoptosis in an orthotopic xenograft NB mouse model. Collectively, our study illustrates the anti-tumor efficacy of ixazomib in NB both alone and in combination with dox, suggesting that combination therapy including ixazomib with traditional therapeutic agents such as dox is a viable strategy that may achieve better outcomes for NB patients.

Neuroblastoma (NB) accounts for about 15% of all pediatric cancer-related mortalities and continues to be the most common malignancy in children^1^. Despite progress in recent decades in development of clinical therapies, the five-year overall survival (OS) rate of high-risk NB patients remains less than 40%^2^. As long as chemotherapy continues to be one of the mainstream clinical modalities used in cancer therapy, chemoresistance will be a recurring problem in populations of cancer cells that survive treatment. Failures in high-risk NB therapy such as relapse and metastasis have been frequently associated with chemoresistance, which is believed to cause over 90% of all failures in metastatic cancer^3^. Thus, a better understanding of the molecular mechanisms of chemoresistance and of overcoming chemoresistance in NB by identifying novel drugs would benefit NB patients and lead to better outcomes.

Doxorubicin (dox) is an FDA-approved chemotherapeutic agent that is widely used in the treatment of a variety of cancer types^4^. Dox causes genotoxic stress in cells by binding and intercalating with DNA to induce reactive oxygen species (ROS)^5^. Dox-induced ROS then activate mitogen-activated protein kinase (MAPK) family members JNK and p38, which trigger dox-induced cytotoxicity by promoting Bax-mediated apoptosis^6^,^7^,^8^. Both JNK and p38 have been reported to play essential roles in cell apoptosis pathways by phosphorylating Bax and facilitating mitochondrial translocation of Bax, which then induces apoptosis^8^. Unfortunately, despite the potent toxicity of dox observed in clinical trials, cancer cells still develop resistance to dox treatment^9^,^10^,^11^,^12^.

Nuclear factor-κB (NF-κB) is a central regulator in responses to multiple stimulations. Its antagonist, IκBα, inhibits NF-κB by masking the nuclear localization signals (NLSs) of NF-κB proteins to keep them in the cytoplasm in an inactive state^13^. NF-κB is known for its central role in immune responses, but it also facilitates chemoresistance and progression of tumors when activated in the presence of most chemotherapeutic agents, including dox^14^,^15^,^16^. Dox-induced NF-κB activation is believed to contribute to the development of chemoresistance of cancer cells exposed to dox treatment^17^,^18^,^19^. Thus, inhibiting NF-κB activity to overcome chemoresistance may be a viable option in cancer therapy.

Ixazomib (MLN9708, trade name Ninlaro), a selective and orally active second-generation proteasome inhibitor, was developed to treat a broad range of cancers^20^ and exhibits anti-tumor efficacy in multiple malignancies^21^,^22^,^23^. To date, the anti-tumor efficacy of ixazomib in NB has yet to be investigated. Here we report that ixazomib suppresses NB cell proliferation and anchorage-independent growth and induces cell apoptosis. Furthermore, in NB cells, including the chemoresistant LA-N-6 cell line, ixazomib synergizes with dox treatment by enhancing the cytotoxicity of dox and overcoming dox resistance by stabilizing the IκBα protein and inhibiting dox-induced NF-κB activation. More importantly, ixazomib potently enhanced dox-induced apoptosis in an orthotopic xenograft NB mouse model. Overall, our study displays the anti-tumor efficacy of ixazomib alone and in combination with dox in NB, illustrating that combination therapy of ixazomib and dox may lead to better outcomes for NB patients.

## Results

### Ixazomib shows cytotoxic effect on a subset of NB cell lines

**Figure 1:**
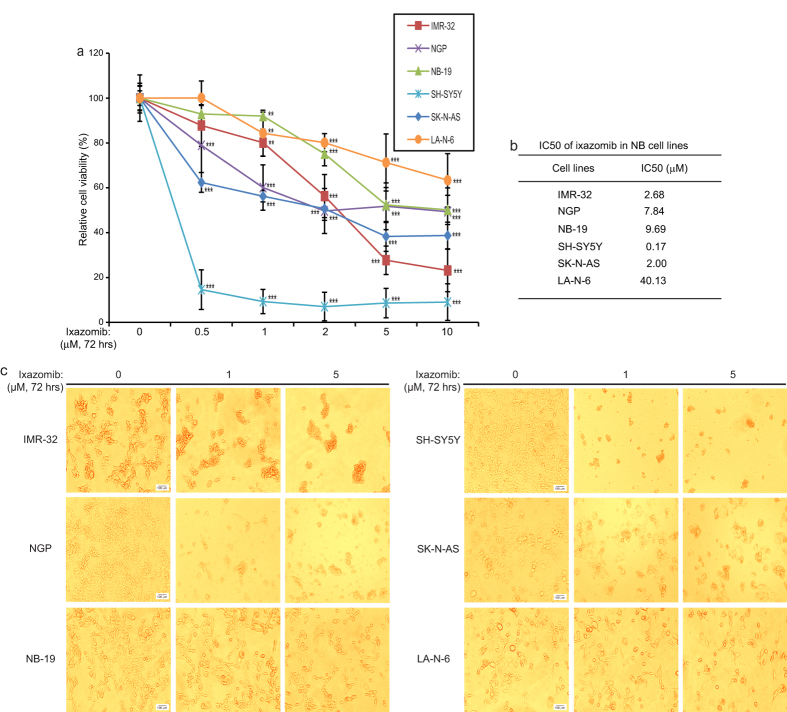
Ixazomib shows cytotoxic effect on NB cells. (**a**) IMR-32, NGP, NB-19, SH-SY5Y, SK-N-AS, and LA-N-6 cells were treated with the indicated concentrations of ixazomib for 72 hrs. Cell viability was then measured with CCK-8 assay, and data was presented as % vehicle ± S.D. *P* < 0.01 (**) or *P* < 0.001 (***) (Student’s t-test, two-tailed) was indicated. (**b**) The IC50 of ixazomib on each NB cell line listed was calculated using Prism 5.0. (**c**) Morphologic changes of the five NB cell lines treated with increasing concentrations of ixazomib for 72 hrs. Scale bars are equal to 100 μm.

To assess the potential cytotoxic effect of ixazomib on NB cells, six human NB cell lines (IMR-32, NGP, NB-19, SH-SY5Y, SK-N-AS and the chemoresistant LA-N-6 cell line) were treated with increasing doses of ixazomib. After 72 hrs of exposure to the inhibitor, the viabilities of the cells were measured. As shown in Fig. 1a, ixazomib suppressed the cell viability of all six NB cell lines tested in a dose-dependent manner. The IC50s of ixazomib in the six NB cells lines were calculated according to the data collected in the cell viability assay (Fig. 1b). Moreover, the cytotoxic effect of ixazomib on the NB cells was further confirmed by cell morphology changes upon treatment (Fig. 1c).

### Ixazomib attenuates the colony formation ability of NB cells

**Figure 2:**
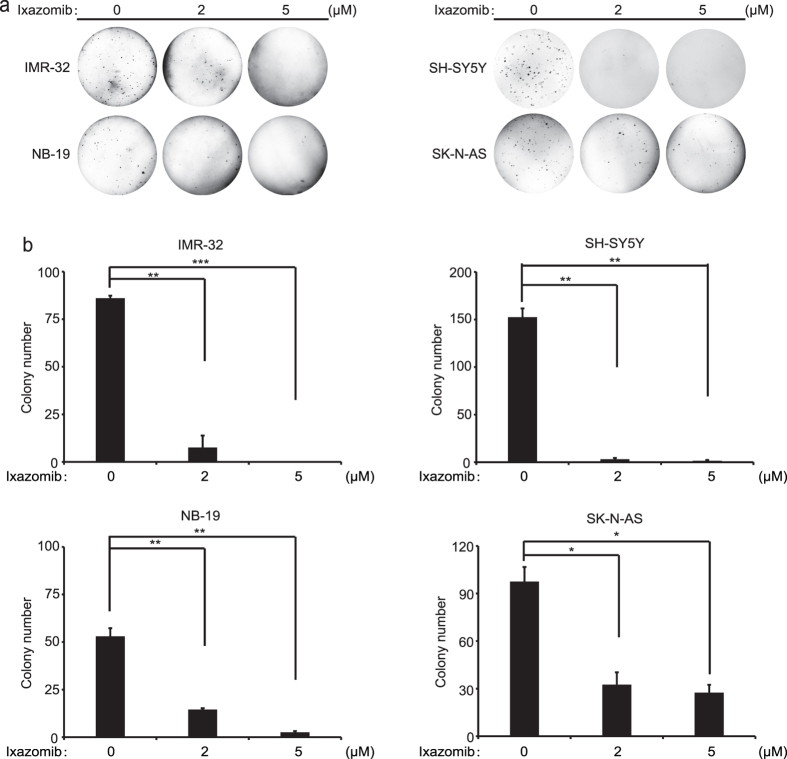
Ixazomib suppresses anchorage-independent growth of NB cells. (**a**) IMR-32, NB-19, SH-SY5Y, and SK-N-AS cells were seeded in six-well plates, treated with the indicated concentrations of ixazomib or with an equal volume of DMSO, and grown for two to three weeks. Cells were stained with 0.005% (w/v) crystal violet for 4 hrs and then pictures were taken. (**b**) Colonies were counted and the results were presented as mean ± S.D. *P* < 0.05 (*), *P* < 0.01 (**), or *P* < 0.001 (***) (Student’s t-test, two-tailed) was shown.

The ability to form colonies in soft agar cultures is one of the defining properties of cancer cells and is frequently used to evaluate the anchorage-independent growth of the cells. To determine whether ixazomib affects the colony formation ability of the NB cell lines, soft agar assays were performed with two concentrations of the inhibitor. As shown in Fig. 2a, ixazomib significantly suppressed the ability of the four chosen NB cell lines to form colonies compared with the control, untreated cells. Colony numbers in the agar were quantified in each group, and we found that ixazomib attenuates the colony formation ability of the tested NB cell lines in a dose dependent manner (Fig. 2b). These results demonstrate that ixazomib significantly inhibits the anchorage-independent growth of a panel of NB cell lines.

### Ixazomib induces apoptosis in most of NB cells

**Figure 3:**
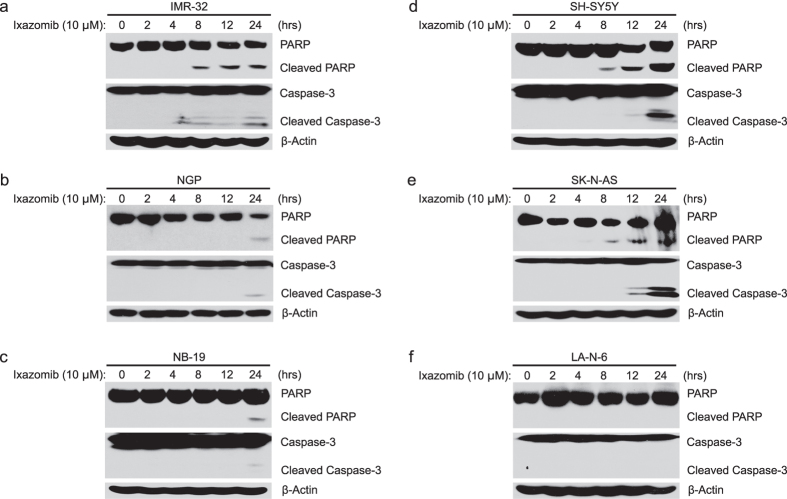
Ixazomib induces apoptosis in most NB cells. (**a–f**) IMR-32 (**a**) NGP (**b**) NB-19 (**c**) SH-SY5Y (**d**) SK-N-AS (**e**) and LA-N-6 (**f**) cells were treated with 10 μM of ixazomib for various time points (0–24 hrs). The cells were then harvested at the end of the treatment, subjected to SDS-PAGE, and immunoblotted with PARP, Caspase-3, and β-actin primary antibodies. β-actin was used as a loading control for whole cell extracts in all samples.

Ixazomib has been reported to induce apoptosis in chronic lymphocytic leukemia (CLL) and multiple myeloma (MM) as a single agent or in combination with other chemotherapeutic agents^21^,^24^. Thus, we hypothesized that ixazomib may induce apoptosis in NB cells. To test our hypothesis, IMR-32, NGP, NB-19, SH-SY5Y, SK-N-AS and LA-N-6 cells were treated with ixazomib at the indicated dose and time points (0–24 hrs). After treatment, cell pellets were collected separately to perform protein immunoblotting assays. As expected, we found that ixazomib induced significant PARP cleavages in IMR-32, SH-SY5Y, and SK-N-AS cells; however, the effect of ixazomib was less significant on NGP and NB-19 cells (Fig. 3a–e). Similar results of Caspase-3 cleavages were obtained in these cell lines upon ixazomib treatment. However, ixazomib alone had little effect on LA-N-6 cells under the same treatment condition as to other NB cell lines (Fig. 3f). These data indicate that ixazomib alone induces apoptosis in the five NB cell lines tested except the chemoresistant LA-N-6 cell line in a time dependent manner.

### Ixazomib augments the cytotoxic effect of dox in NB cells

**Figure 4:**
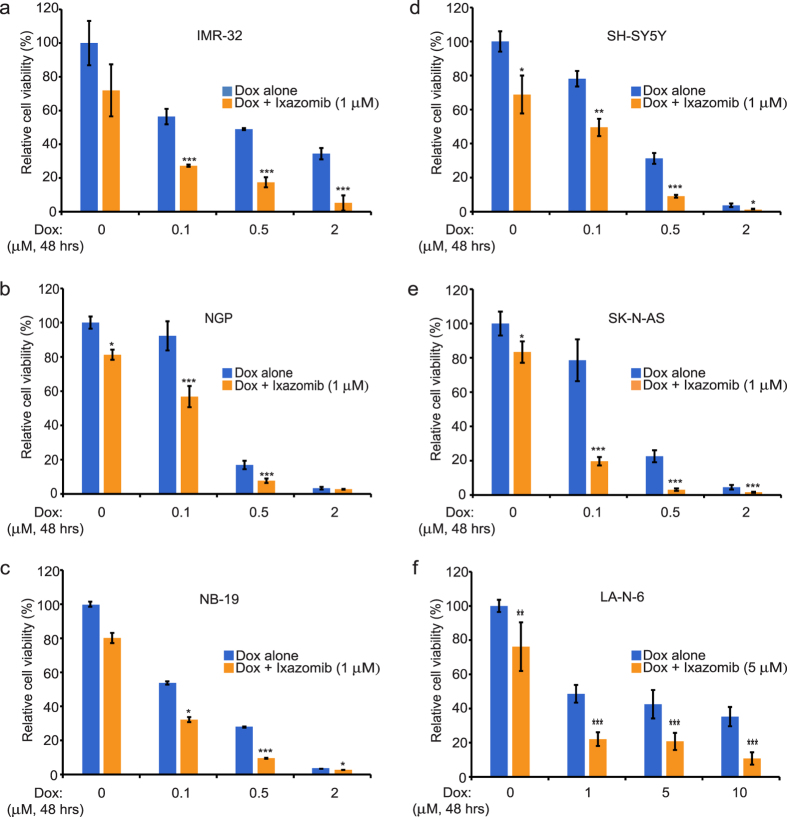
Ixazomib enhances dox-induced cytotoxicity in a group of NB cell lines. (**a–f**) IMR-32 (**a**) NGP (**b**) NB-19 (**c**) SH-SY5Y (**d**) SK-N-AS (**e**) and LA-N-6 (**f**) cells were seeded in 96-well plates and incubated with the indicated concentrations of dox plus DMSO or ixazomib (1 μM or 5 μM) for 48 hrs. Cell viability was then measured by CCK-8 assay. Results were presented as mean ± S.D. *P* < 0.05 (*), *P* < 0.01 (**) or *P* < 0.001 (***) (Student’s t-test, two-tailed) was indicated.

Dox induces apoptosis in tumor cells by causing DNA damage^25^, and ixazomib has been shown to induce apoptosis in CLL cells in combination with other agents^24^. Therefore, we hypothesized that inhibition of proteasome activity by ixazomib may increase the chemosensitivity of NB cells to dox. To test our hypothesis, we treated IMR-32, NGP, NB-19, SH-SY5Y, SK-N-AS and LA-N-6 cells with either dox alone or in combination with ixazomib (1 μM or 5 μM). As displayed in Fig. 4a–f, the combination of ixazomib with dox shows a greater inhibitory effect on cell proliferation than single agent dox treatment. These results suggest that ixazomib sensitizes a subset of NB cell lines to dox treatment, therefore augmenting the cytotoxic effect of dox in NB cells.

### Ixazomib enhances dox-induced apoptosis both *in vitro* and *in vivo*

**Figure 5:**
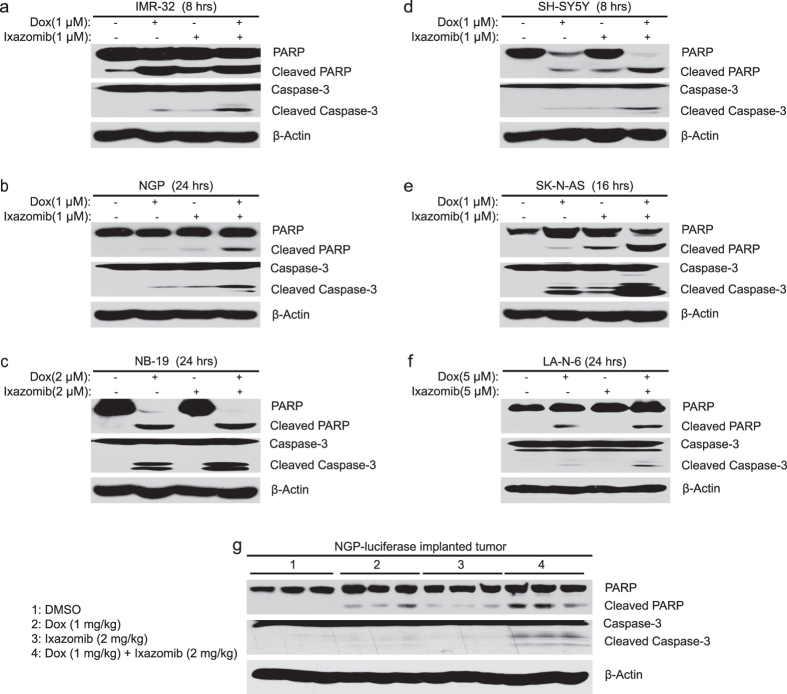
Ixazomib enhances dox-induced apoptosis in NB cells and in an orthotopic xenograft NB mouse model. (**a**–**f**) IMR-32 (**a**) NGP (**b**) NB-19 (**c**) SH-SY5Y (**d**) SK-N-AS (**e**) and LA-N-6 (**f**) cells were treated with dox alone (1 μM or 2 μM or 5 μM), ixazomib alone (1 μM or 2 μM or 5 μM), or their combinations for different time points (8–24 hrs), and the cells were collected, subjected to SDS-PAGE, and immunoblotted with PARP, Caspase-3, and β-actin primary antibodies. (**g**) The mice bearing NGP xenografted tumors for six weeks were treated as described, and then the tumors were harvested and lysed, subjected to SDS-PAGE and immunoblotted with the indicated antibodies. β-actin was used as a loading control for whole cell extracts in all samples.

To determine whether ixazomib could enhance dox-induced apoptosis in NB cells, six NB cell lines were treated with dox alone (1 μM or 2 μM or 5 μM), ixazomib alone (1 μM or 2 μM or 5 μM), or their combination for the indicated time points. The cells were then collected for the immunoblotting assay. Compared with the single agent treatment groups with either dox or ixazomib, the combination group showed stronger PARP and Caspase-3 cleavages in all six of the cell lines tested (Fig. 5a–f), suggesting that ixazomib enhances dox-induced apoptosis in NB cells.

To determine the anti-tumor efficacy of ixazomib *in vivo*, the NGP-luciferase cells xenografted nude mice were treated with dimethyl sulfoxide (DMSO) or dox (1 mg/kg) or ixazomib (2 mg/kg) or their combination by intraperitoneal (i.p.) injection daily for four days. And then the tumors were harvested and lysed for protein immunoblotting. As shown in Fig. 5g, the combination group of dox and ixazomib treatment significantly increased PARP and Caspase-3 cleavages compared with DMSO control group or the single agent treatment groups with either dox or ixazomib, indicating that ixazomib augments dox-induced apoptosis in an orthotopic xenograft NB mouse model. Taken together, ixazomib enhances dox-induced apoptosis both *in vitro* and *in vivo*.

### Ixazomib enhances dox-induced p38 and JNK activity and decreases dox-induced IκBα degradation

**Figure 6:**
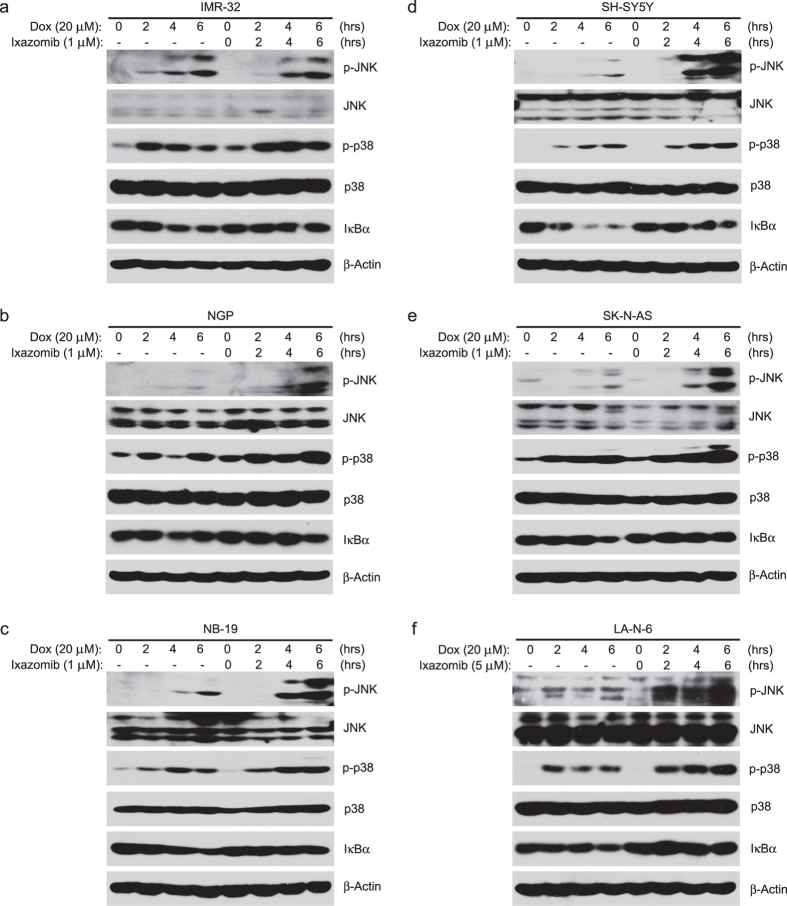
Ixazomib sensitizes NB cells to dox-induced apoptosis by upregulating dox-induced p38 and JNK activity and by decreasing dox-induced IκBα degradation. (**a**–**f**) IMR-32 (**a**) NGP (**b**) NB-19 (**c**) SH-SY5Y (**d**) SK-N-AS (**e**) and LA-N-6 (**f**) cells were exposed to 20 μM dox with or without ixazomib (1 μM or 5 μM) for 0–6 hrs, respectively. The cells were then harvested and lysed for immunoblotting with the indicated antibodies. β-actin was used as a loading control.

The activation of MAPK members JNK and p38 has been reported to be responsible for dox-induced apoptosis^6^,^26^,^27^. To determine the mechanism of the enhanced cytotoxic effect of ixazomib on dox treated NB cells, we treated the NB cells with dox (20 μM) alone or in combination with ixazomib (1 μM or 5 μM) at different time points. After performing the protein immunoblotting assay, we found that the combination treatment increased the phosphorylation of p38 and JNK compared to the dox single treatment (Fig. 6a–f).

**Figure 7:**
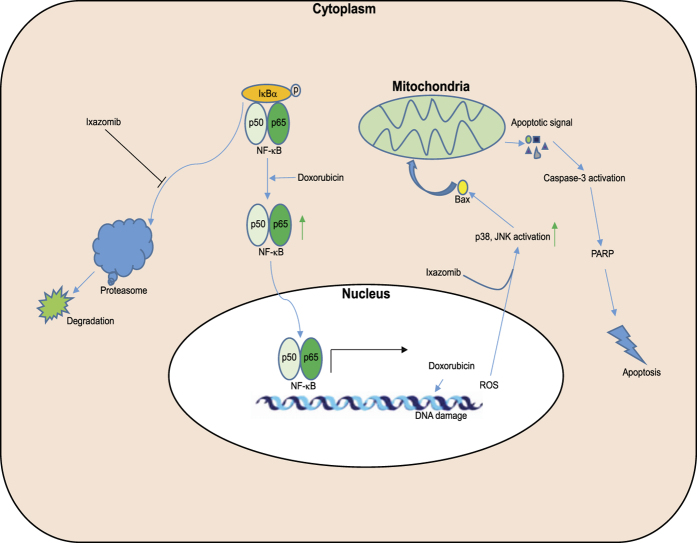
The working model for proteasome inhibitor ixazomib in dox-induced NB cell apoptosis. Proteasome inhibition by ixazomib upregulates dox-induced p38 and JNK activity to augment dox-induced apoptosis. ixazomib also inhibits dox-induced IκBα degradation, thus attenuating NF-κB mediated dox resistance in NB cells.

Previous studies have also shown that dox treatment in MCF-7 cells caused drug resistance in a NF-κB dependent manner^28^ and that the inhibition of NF-κB activity could at least partially overcome dox-induced resistance in MCF-7 and human osteosarcoma cell lines^19^,^29^. IκBα is known to negatively regulate NF-κB by blocking the NLS of the NF-κB protein to keep it sequestered in an inactive state in the cytoplasm^13^. Thus, to determine whether the sensitization effect of ixazomib on dox-induced cytotoxicity results from the inhibition of NF-κB activity, IκBα protein level was tested. As illustrated in Fig. 6a–f, dox induced IκBα protein degradation whereas ixazomib suppressed dox-induced IκBα protein degradation in all six NB cell lines tested, suggesting that dox-induced NF-κB activity is inhibited by ixazomib. Taken together, our results support a working model in which ixazomib enhances dox-induced apoptosis by suppressing dox-induced NF-κB activity (Fig. 7).

## Discussion

Consisting of multiple proteases, the proteasome is an essential component of the ubiquitin-proteasome system, which is responsible for the degradation of intracellular proteins^30^. The high abundance and ubiquitous presence of proteasomes in the cytosol reveals the central role of proteasomes played in the regulation of cell cycle control, apoptosis, cellular stress response, and cell fate determination^31^. Dysfunction of the proteasome is associated with tumor cell survival, and pharmacological inhibition of proteasome activity by small molecule inhibitors shows anti-tumor efficacy in various cancer types^32^,^33^,^34^,^35^,^36^. Here we report that the second generation proteasome inhibitor ixazomib exerts its anti-tumor effect in NB by suppressing NB cell proliferation and inducing apoptosis.

As an anthracycline antitumor antibiotic, dox intercalates within the double strands of DNA and causes DNA damage, thus inhibiting the progression of topoisomerase II. This, in turn, relaxes supercoils in DNA for transcription and ultimately results in apoptosis^37^. Dox has been used as an anti-cancer agent for treating a variety of cancer types. However, clinical application of high dose of dox is limited due to its association with multiple damaging side effects, especially injury to the heart^5^. Thus, finding ways to enhance the intended anti-cancer effects of dox is vitally important. In this study, we show that ixazomib significantly enhances the cytotoxicity of dox in NB cells. Moreover, ixazomib augments dox-induced apoptosis by promoting JNK and p38 mediated apoptosis.

Chemoresistance has been considered as one of the major causes of relapse in patients, especially in high-risk NB patients. Therefore, clarifying the molecular mechanisms that are responsible for chemoresistance is of vital importance. Activation of the transcription factor NF-κB has been reported to be a mechanism for chemoresistance and, thus, targeting NF-κB pathway to overcome chemoresistance may be a viable strategy^38^. The proteasome has been reported to be involved in NF-κB activation by promoting the degradation of its inhibitor IκBα^39^. Therefore, pharmacological inhibition of proteasome activity results in attenuated dox-induced chemoresistance. We found that ixazomib could overcome dox-induced chemoresistance by stabilizing IκBα expression levels and inactivating NF-κB in NB cell lines, including the chemoresistance LA-N-6 cell line. Therefore, ixazomib improves the therapeutic index of dox and broadens the spectrum of dox applications.

Due to the critical roles that the proteasome plays in cellular functions, aberrant activation or deactivation of proteasomes may affect a variety of proteins and result in many human diseases, including cancer^40^. The orally active proteasome inhibitor ixazomib has been approved by the FDA for the treatment of patients with multiple myeloma in combination with lenalidomide and dexamethasone^41^. Compared with the first generation proteasome inhibitor bortezomib, ixazomib exhibits better clinical outcomes and reduced toxicities in the treatment of patients with multiple myeloma, due to modified chemical moieties. Therefore, it’s likely that ixazomib may achieve better outcome for NB patients.

In summary, our study reveals the anti-tumor efficacy of ixazomib in NB cells. In NB cells, ixazomib exerts potent anti-oncogenic effects not only by inducing apoptosis but also by enhancing the cytotoxicity of dox. Moreover, ixazomib sensitizes NB cells, including the chemoresistant LA-N-6 cells, to dox treatment by stabilizing IκBα and suppressing dox-induced NF-κB activity. More importantly, ixazomib potently enhanced dox-induced cellular apoptosis of the tumor cells in an orthotopic xenograft NB mouse model. Therefore, it’s feasible to initiate clinical trials for the treatment of NB by including ixazomib in combination with current therapeutic agents like dox. This study supports the idea that combination therapies of proteasome inhibitors with standard chemotherapeutic agents will achieve better outcomes in NB therapy.

## Materials and Methods

### Antibodies and Reagents

The proteasome inhibitor ixazomib was purchased from APExBIO (A4007) (Houston, TX, USA). Doxorubicin (dox, D1515) and anti-β-Actin (A2228) antibody were purchased from Sigma (Sigma-Aldrich Corp, St. Louis, MO, USA). Anti-PARP (9532S), anti-Caspase-3 (9662S), anti-phospho-SAPK/JNK (Thr183/Tyr185) (9251S), anti-SAPK/JNK (9258S), anti-phospho-p38 MAP kinase (Thr180/Tyr182) (9211S), anti-p38 MAP kinase (8690S), anti-IκBα (9242S), anti-rabbit (7074S) IgG, and anti-mouse (7076S) antibodies were purchased from Cell Signaling Technology (Cell Signaling Technology, Danvers, MA, USA).

### Cell Lines and Cell Culture

Human NB cell lines IMR-32, NGP, NB-19, SH-SY5Y, and SK-N-AS were cultured in Roswell Park Memorial Institute (RPMI) medium 1640 (Lonza, Walkersville, MD, USA), which was supplemented with 10% (v/v) heat-inactivated Fetal Bovine Serum (FBS) (Sigma-Aldrich Co. LLC. St. Louis, MO, USA), 100 μg/mL streptomycin, and 100 units/mL penicillin. The chemoresistant NB cell line LA-N-6 was grown in RPMI containing 20% (v/v) heat-inactivated FBS, 100 units/mL penicillin, and 100 μg/mL streptomycin. The NB-19 cell line was provided by Dr. Andrew M. Davidoff (St. Judes’s Children’s Research Hospital, Memphis, TN, USA) and LA-N-6 cell line was provided by Dr. Robert C. Seeger (Children’s Hospital of Los Angeles, Los Angeles, CA, USA). The NGP-luciferase cell line was generated by transfection with a pcDNA3 luciferase expression plasmid into the NGP cells. A stable cell line was established after 800 μg/ml of G418 (Enzo Life Sciences, Farmingdale, NY, USA) selection for 10 days. All the other cell lines were obtained from the American Type Culture Collection (ATCC, Manassas, VA, USA). All cells were kept in a humidified incubator at 37 °C with 5% CO_2_.

### Cell Viability Assay

Cell Counting KIT-8 (CCK-8, WST-8[2-(2-methoxy-4-nitrophenyl)-3-(4-nitrophenyl)-5-(2,4-disulfophenyl)-2 H-tetrazolium, monosodium salt]) (Dojindo Laboratories, Rockville, MA, USA) was used to measure the viability of cells after treatment with ixazomib, dox, or a combination of both. Briefly, cells were seeded in 96-well plates at 5 × 10^3^ cells per well. After growing for 24 hrs, the cells were exposed to fresh medium or increasing doses of ixazomib, dox, or the combination at 37 °C for 48 or 72 hrs. Then, a mixture of 10 μL of CCK-8 and 190 μL of RPMI with 10% FBS was added into each well. The absorbance of each well was detected by using a microplate reader at 450 nm four hrs later. Each experiment was performed in three or six replicates and the background reading of the media was subtracted from each well to standardize the readings.

### Cell Imaging

Six NB cell lines were seeded in 96-well plates at 5 × 10^3^ cells per well. The cells were treated with the indicated concentrations of ixazomib, dox, or the combination, or equal volumes of DMSO for 48 hrs or 72 hrs. Cell morphologies were then captured using an optical microscope.

### Colony Formation Assay

The colony formation assay was performed as previously described^42^,^43^. To make a 5% (w/v) base agar, agar powder (214220, Difco Laboratories, Detroit, MI, USA) was added to distilled water, and the mixture was autoclaved for 50 min. The mixture was then cooled down in a 56 °C water bath. For the bottom agar layer, each well in the six-well plate was filled with 2 mL of the 0.5% agar/RPMI 1640 (supplemented with 10% FBS) solution, and the solution was cooled until semi-solid. For the top agar layer, 8 × 10^3^ pre-counted NB cells per well were mixed with 1.5 ml of 0.3% agar. Indicated concentrations of ixazomib were added to the wells 24 hrs later. Cells were cultured at 37 °C for two to three weeks and then stained with 500 μL of 0.005% crystal violet (C3886, Sigma). Four hours later, cell images were taken and the colonies were counted by using the VersaDoc Imaging System (Bio-Rad Laboratories, Hercules, CA, USA). All the assays were performed in triplicate, and representative images were shown in the figures.

### Protein Immunoblotting

The protein immunoblotting assay was performed as described^44^,^45^. Cells were collected at the end of the treatments and lysed for 30 min at 4 °C in cooled RIPA buffer (50 mM Tris-HCl at pH 7.4, 150 mM NaCl, 1 mM EDTA, 1% NP-40, 0.25% sodium deoxycholate, 1 mM phenylmethylsulfonyl fluoride, 1 mM benzamidine, 10 μg/mL leupeptin, 1 mM dithiothreitol, 50 mM sodium fluoride, 0.1 mM sodium orthovanadate, and phosphatase inhibitor cocktail 2 and 3 (p5726 and p0044, Sigma)). The cell lysates were centrifuged at 13,000 rpm for 15 min, and the supernatants were used as loading samples. Protein concentrations of the samples were measured using a Bradford reagent (Bio-Rad Laboratories, Hercules, CA, USA), and each sample was mixed with 4 × loading buffer before heating. The mixture was then heated at 100 °C for 8 min to denature the samples. Then, the samples were separated by SDS-PAGE, transferred to polyvinylidence fluoride (PVDF) membranes (EMD-Milipore, Billerica, MA, USA), blocked with 5% milk for one hour at room temperature (RT, 25 °C), and probed with the suggested dilutions of indicated primary antibodies overnight at 4 °C. Anti-mouse or rabbit secondary antibodies conjugated with horseradish peroxidase were incubated with the membranes at RT for one hour. After that, the signals on the membranes were detected using the ECL-Plus Western Detection System (GE Health Care, Buckinghamshire, UK). β-actin was used as a loading control for whole cell extracts in all groups.

### Combinatorial effect of ixazomib and dox in an orthotopic xenograft NB mouse model

Female athymic NCR nude mice of five to six-week-old were purchased from Taconic (Taconic, Hudson, NY, USA) and kept under barrier conditions (pathogen-free conditions provided by plastic cages with sealed air filters). The orthotopic xenograft NB mouse model was established by using orthotopic (intrarenal) implantation of the NB cells as described previously^46^,^47^. Briefly, a transverse incision was created over the left flank of the nude mice and 1.5 × 10^6^ luciferase-transduced NGP cells in 0.1 ml of PBS were surgically injected into the left renal capsule of the left kidney of the nude mice.

Six weeks after implantation, the NGP-luciferase cells implanted nude mice were treated with DMSO or dox (1 mg/kg) or ixazomib (2 mg/kg) or their combination by i.p. injection daily for four days. At the end of the treatment, the mice were sacrificed and the tumors were harvested and lysed for protein immunoblotting. All mice were handled according to protocols approved by the Institutional Animal Care and Use Committee of the Baylor College of Medicine.

### Statistical Analysis

All the presented values were shown as mean ± S.D. A two-tailed Student’s t-test was used to determine the statistical significance of all the assays between the control and drug treated groups. A *P-*value < 0.05 was considered to be statistically significant in all of the assays. Each assay was performed at least twice and the representative results were presented in the figures.

## Additional Information

**How to cite this article**: Li, H. *et al.* Novel proteasome inhibitor ixazomib sensitizes neuroblastoma cells to doxorubicin treatment. *Sci. Rep.* **6**, 34397; doi: 10.1038/srep34397 (2016).
